# Deciphering the complexity of enteric niches in Hirschsprung disease: from metaphorical insights to therapeutic transformation

**DOI:** 10.3389/fped.2025.1579290

**Published:** 2025-06-19

**Authors:** Hui Yu, Weikang Pan, Donghao Tian, Ya Gao

**Affiliations:** Department of Pediatric Surgery, The Second Affiliated Hospital, Xi’an Jiaotong University, Xi’an, China

**Keywords:** Hirschsprung disease, enteric niches, enteric nervous system, neurogenesis HSCR, microbial metabolism, immune modulation, disease pathogenesis “enteric niches” AND (“gut microbiome” OR “host-pathogen interactions”)

## Abstract

Hirschsprung disease (HSCR) is a congenital disorder marked by the absence of ganglion cells in the distal bowel, resulting in severe constipation and bowel obstruction. Surgery is currently the primary treatment for HSCR. However, post-surgical outcomes are unsatisfactory, merely alleviating symptoms of intestinal obstruction. Up to one-third of HSCR patients continue to experience gastrointestinal issues post-surgery, which severely impacts their growth and development and may even threaten their lives. Cell transplantation represents a promising strategy for the radical treatment of HSCR but faces numerous challenges. The mismatch between transplanted cells and the enteric niches is one of the key obstacles in reconstructing the enteric nervous system through cell transplantation. There is an urgent need to “get to the root of the problem” to enhance our understanding of the enteric niches and overcome current limitations. This review synthesizes insights from two metaphorical narratives, “Blind Men and the Elephant” and “Rags To Riches,” to provide a comprehensive re-understanding of the enteric niches and their potential for enteric neurogenesis. By examining these narratives, we underscore the importance of the enteric niches in the potential for transformative discoveries in HSCR research.

## Graphical Abstract

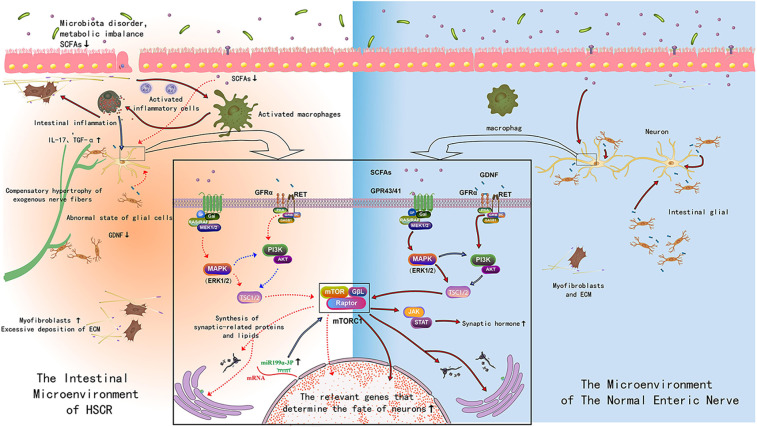

## Introduction

Surgical resection to remove the aganglionic portion of the gut is currently the main treatment method for treating Hirschsprung disease (HSCR). However, the surgical outcome of patients is unsatisfactory, with up to one-third of patients suffering from persistent dysfunctions, such as enterocolitis and incontinence after surgery, which affect the growth and development of affected children (1). This surgical treatment has transformed HSCR from a life-threatening defect into a long-term chronic disease. With the current rapid development of translational medicine, enteric neural crest-derived cells (ENCCs) are now recognized to possess “stemness,” namely, with the potential of self-renewal and differentiation to form effective neural networks, and their transplantation can partially restore intestinal functions (2). Cell transplantation of ENCCs has been considered as one of the new therapeutic strategies and frontier research hotspots for HSCR. Our research findings include optimizing cell culture systems, developing intestinal organoids, and enhancing cell phenotype potential with drugs such as mosapride, retinoic acid, endothelin-3, glycogen synthase kinase inhibitors, and granulocyte colony-stimulating factor (3). Despite this, there is still a large gap between current research progress and clinical transformation. Many scholars believe that the correct development and maturation of the enteric nervous system (ENS) are essential conditions, not achieved until now, for the treatment of HSCR with ENCCs, which depends on a series of highly coordinated events in a spatiotemporal order, such as sufficient proliferation, directional migration, orderly differentiation of the ENCCs, and appropriate neurochemical phenotypes, sufficient axonal extension, and effective synaptogenesis (4). These events are closely related to the enteric niches.

### Literature review algorithm

#### Scope definition focus: enteric niches in host-microbe interactions

Key themes: HSCR, Microbial metabolism, immune modulation, disease pathogenesis.

#### Search strategy databases: PubMed, web of science, scopus

Keywords: “enteric niches” AND (“gut microbiome” OR “host-pathogen interactions”)

## Filters: experimental/clinical studies

### Aim of the review

This review underscores the challenges in HSCR treatment, emphasizing the critical role of enteric niches - a complex microenvironment comprising genomic factors, microbiota, fibroblasts, ECM, immune interactions, and smooth muscle cells. While deciphering disease-associated mutations remains essential, a holistic approach is required to address niche heterogeneity and enhance the efficacy of cell transplantation therapies.

### Insights from “blind Men and the elephant”

The enteric niches are composed of complex microenvironments, which can be divided into resident cells, their metabolism, and so on. The enteric niches provide fundamental support for the development and maturation of the ENS, which are inseparable and interact with each other. Under the spatiotemporal coupling of specific morphogenic and directional factors from enteric niches, ENCCs initiate self-proliferation and ordered migration, differentiate into neurons and glia, and construct a functional neural network (5). Meanwhile, the surface receptors and intracellular signal transduction pathways of ENCCs also change and participate in the remodeling of enteric niches (6, 7).

In reality, the enteric niches in patients with HSCR harbor numerous abnormalities, encompassing the physical and resident cell niches, as well as the niches of the gut microbiota and their metabolisms. The detrimental impact of these abnormalities on cell transplantation therapy for HSCR has been significantly underestimated and remains inadequately explored. Few studies have thoroughly examined the role of these enteric niches in this context.

The physical niches, which form a dynamic three-dimensional ultrastructure, are composed of a variety of extracellular matrix components. These components are essential for the structural integrity and function of the ENS. The resident cellular niches are inhabited by an array of cell types, such as intestinal mesenchymal cells, glial cells, smooth muscle cells, Interstitial cells of Cajal (ICC), intestinal epithelial cells, and immune cells. These cells play a critical role in the maintenance and regulation of the ENS, contributing to its normal development and function through the secretion of key cytokines and other factors that influence the behavior of ENCCs (8). In a cohort of patients with common HSCR, variations in genes involved in cell-adhesion proteins, such as integrin beta-4 and focal adhesion kinase, have been detected (9). Histological evidence also suggests abnormal physical niches in HSCR patients and animal models, characterized by high expression and abnormal distribution of components like hyaluronic acid, collagen, laminin, fibronectin, and cellular adhesion molecules, leading to delayed migration and failed colonization of ENCCs (10, 11). Our studies have shown that the proliferation activity of intestinal mesenchymal cells is reduced in HSCR mouse models, affecting the normal settlement and migration of ENCCs (12). HSCR patients also exhibit transcriptional abnormalities in the GDNF gene, with decreased expression of GDNF protein/gene and a reduced number of GDNF immunoreactive cells (13, 14). Smooth muscle cells in HSCR patients typically exhibit hypertrophy, an increased number of actin and myosin filaments, and an excessive response to neurotransmitters such as acetylcholine and norepinephrine (15, 16). Changes in the expression of cytoskeletal proteins and *ε*-myosin complexes are observed, with a loss or significant reduction in their expression levels (15, 16). Abnormally elevated expression of Actin Alpha 2 in the circular smooth muscle of the aganglionic segment leads to hyperactive contraction (17). In HSCR patients, the denervated segment exhibits a decrease or absence of ICC density, and spatial differences in intermuscular and submucosal ICC (18, 19). Relevant ultrastructural studies show that ICC has smaller volume and abnormal shapes, and a decrease in organelles such as mitochondria and endoplasmic reticulum in the aganglionic segment (18). Multiple intestinal epithelial cell subsets, such as goblet and enteroendocrine cells, undergo significant changes in the aganglionic segments of HSCR patients and animal models (19). Goblet cells show an increased cell number and volume, decreased intracellular mucin, and differential expression of MUC2, TFF3, SPDEF, and KLF4 compared to normal intestinal segments. Functionally, they exhibit higher epithelial resistance. Similarly, the number of enteroendocrine cells positive for synaptogenin, PYY, somatostatin, and 5-HT is increased in the aganglionic segments of common and long-segment HSCR (19). In addition, the HSCR animal model attained after treated with benzalkonium chloride also showed an increase in the number of enteroendocrine cells (20). The intestinal mucosa of HSCR patients and animal models showed immune abnormalities, with a reduced number of lymphocyte in the gut-associated lymphoid tissue Peyer's patches, particularly in mature IgM^+^ IgDhi B-lymphocytes, as well as reduced number and proportions of IgA cell (21). Additionally, structural changes such as decreased spleen volume, decreased red pulp, and a lack of B-lymphocytes in the germinal centers and marginal zones are observed. Furthermore, there is a decrease in the expression of ATP-sensitive K^(+)^ channels (22). In HSCR animal models an increase in the number of ED2-positive macrophages in the muscle interstitium of the denervated segment was observed (23). Finally, abnormal increase and overactivation of mast cells exists in the submucosa, lamina propria, and muscles of the denervated segments were found in children with HSCR, leading to inflammation and inflammation-mediated neuronal apoptosis (24, 25). The expression of pro-inflammatory cytokines IL-17 and IL-36*γ* are significantly increased in HSCR patients (26, 27), while the expression of caveolin-1, a key factor of the intestinal epithelial barrier, is significantly decreased (28). The expression of various inflammatory factors, such as C-reactive protein, tumor necrosis factor-ɑ, and interleukin-17, are significantly increased in our HSCR animal models (29). Some studies defined inflammation as more than 10 IgG-positive cells in a region of 100 IgA cells and found that nearly half of children with HSCR had similar intestinal inflammation in aganglionic and normal intestinal segments (30).

Furthermore, in both HSCR patients and animal models, there is evidence of intestinal dysbiosis and the presence of abnormal metabolites. This dysbiosis is characterized by a reduced overall abundance of microbial species, and notable differences in the presence of characteristic flora (31, 32). Additionally, there is a decrease in the production of short-chain fatty acids (31, 32), which are critical for the health and function of the gut and its associated ENS. These alterations in the gut microbiota and their metabolites may have implications for the efficacy of therapeutic interventions, such as cell transplantation.

It should be noted that there is no strict distinction among the above physical and resident cell niches, as well as the niches of the gut microbiota and their metabolisms, but they are interrelated, interdependent, and mutually influential to jointly construct an organic whole with specific functions. For example, resident cell niches secrete various extracellular matrix components and actively release GDNF and endothelin-3 to form the physical scaffold that provide physical support for the three-dimensional structure of the gut and chemical directional spatiotemporal induction for the self-proliferation, orderly migration, and directed differentiation of ENCCs.

The parable of the “Blind Men and the Elephant” serves as a metaphor for the necessity of adopting a holistic approach when investigating the intricate nature of enteric niches. In the context of HSCR, each blind man symbolizes a distinct facet of the enteric niches, including the physical and resident cellular components, as well as the gut microbiota and their metabolic activities. By synthesizing these varied viewpoints, we aim to achieve a more complete, or “elephantine,” understanding of the multifaceted role that enteric niches play in the treatment of HSCR, which is crucial for advancing our knowledge and developing more effective therapeutic strategies.

### Insights from “rags to riches”

The aforementioned abnormalities within the enteric niches frequently present challenges for cell transplantation therapy in HSCR. It is evident that the excessive formation and uneven distribution of the extracellular matrix can impact the colonization and migration of ENCCs, primarily affecting their differentiation post-transplantation (33, 34). The “untimely” activation of laminin receptors in ENCCs results in premature exit from the cell cycle and entry into the differentiation phase upon transplantation, which further impedes the normal colonization and migration of ENCCs (35, 36). Additionally, abnormal enteric niches can alter the direction of ENCCs differentiation and enhance neural differentiation during transplantation (34, 37). Overexpression of fibronectin can cause sustained damage to the progeny cells of ENCCs, significantly reducing the expression of excitatory and inhibitory synaptic markers and affecting the excitatory/inhibitory synaptic function in HSCR (38).

Moreover, the impact of intestinal inflammation derived from dysbiosis, on transplanted ENCCs and their differentiated offspring cells must be meticulously considered. For instance, intestinal inflammation can negatively regulate ENCCs and their progeny by elevating the levels of nitric oxide produced from inducible nitric oxide synthase (39). It can also induce neuronal apoptosis through rapid inhibition of mitochondrial respiration, slow inhibition of glycolysis, induction of mitochondrial permeability transition, activation of poly-ADP-ribose polymerase, oxidative activation of p53, p38 MAPK pathway, or endoplasmic reticulum stress (40).

Some studies have implemented corresponding strategies to address these abnormal enteric niches, achieving a certain level of therapeutic efficacy. The optimization of these abnormalities in the enteric niches has emerged as a highly promising therapeutic target for cell transplantation in the treatment of HSCR. Based on the absence of native directional synergistic cues, which can lead to abnormal aggregation and disordered migration of transplanted cells, tissue engineering employs biomimetic and bioactive materials, such as nanofibers, treated with a variety of extracellular matrix-derived bioactive peptide epitopes. These materials are designed to guide the directional migration of transplanted cells and facilitate the orderly connection of axons. This approach aims to reduce the abnormal aggregation of cells, increase neural dendrites, and promote the electrophysiological maturation of neurons (41–43). Additionally, pretreatment with collagenase and fibronectin can induce ENCCs to migrate towards the distal part of the intestinal lumen, thereby improving the efficiency of cell transplantation (44). Furthermore, various combination methods have been explored. For instance, the double transgenic intervention of GDNF and its receptor GFRα-1 in rat bone marrow mesenchymal stem cells (MSCs) has been studied. The results indicated that these treated MSCs were capable of colonizing and surviving, expressing related genes, and partially restoring the function of colon peristalsis (45). Research has also demonstrated that ICC can cooperatively promote cell transplantation for the treatment of HSCR. Co-transplantation of ENCCs and ICCs can accelerate neurogenesis and enhance intestinal peristalsis in a rat model (46). Lastly, endothelial cells have been shown to promote ENCCs migration and proliferation after transplantation through the β1 integrin signaling pathway (47).

Although the pathogenic mechanisms of HSCR are relatively well established, and radical resection of the aganglionic segment is commonly adopted, the curative effects have not been fully satisfied. This suggests that there may be other potential causes that need to be further explored. Interestingly, in the animal model of HSCR, there were other congenital defects in the proximal “normal” segment of the intestine. The ratio of excitatory motor neurons was greater in the model, and the number of glial cells increased closer to the aganglionic area (48). Additionally, the ganglion volume and neuronal density decreased, and the expression of neurotransmitters changed, with a decrease in the proportion of nNOS and VIP positive neurons and an increase in the proportion of ChAT positive neurons (49). Compared with the healthy group, there were transcriptional differences between the aganglionic and adjacent ganglionic segments in HSCR. These differences included upregulation of genes related to inflammation, cell differentiation, and proliferation, as well as downregulation of genes encoding mucins (50). Additionally, the proximal ganglionic segment in HSCR exhibits similarities to derived organoids in terms of epithelial cell differentiation, epithelial barrier formation, and response to bacterial metabolites and proinflammatory cytokine stimulation (50). This suggests that the proximal ganglionic segment still has abnormalities, such as gene expression disorder, abnormal function, and excessive stress. Furthermore, histopathological abnormalities, such as intestinal neuronal dysplasia-like submucosal ganglion cell hyperplasia and lower histopathological grade of ganglion cells (35, 51), are present in the proximal resected bowel in HSCR. These abnormalities are positively correlated with postoperative incontinence and constipation. As Heather discussed, all is not normal in “normoganglionic” bowel regions of HSCR model (36), let alone aganglionic segments. Therefore, it is of great practical significance to further our understanding of the pathological changes in the proximal ganglionic segment for HSCR. Currently, intra-operative and post-operative pathology focuses on the presence or absence of neurons and cytoplasmic markers, as well as nuclear histopathological characteristics (52). This approach is somewhat insufficient, akin to the parable of the “Blind Men and the Elephant,” where individual components are investigated but not how these components fit together to describe the overall pathology. Further refined evaluation of neuronal/glial subtypes and their proportion, neurotransmitters, and intestinal mucosa is needed.

Fortunately, Our studies have identified that GDNF, short-chain fatty acids (SCFA), and other pleiotropic factors can stimulate neurogenesis and optimize the enteric niches. GDNF can not only promote neurogenesis but also significantly reduce epithelial permeability, smooth muscle thickness, and neutrophil density, while increasing beneficial populations of intestinal flora (53). Another factor, SCFA, not only alleviates intestinal inflammation but also enhances neurogenesis and improves cell transplantation through the MEK1/2 signaling pathway (54). Although GDNF, SCFA, and other pleiotropic factors have strong potential for transforming the enteric niches and stimulating neurogenesis, it is still necessary to further verify whether these pleiotropic factors can repair all the other abnormalities in HSCR patients. This verification is essential to determine their efficacy and applicability in clinical settings.

It is worth considering whether we can do “Rags To Riches” and make full use of the aberrant enteric niches in HSCR. In addition to the absence of enteric ganglia, another pathological feature of HSCR is compensatory hypertrophy of exogenous nerve fibers. The transition zone in HSCR patients provides a good window for understanding the potential correlation between enteric ganglia and exogenous nerve fibers. At the same spatiotemporal point, the transition zone is characterized by reduced enteric ganglia and increased density of exogenous nerve fibers, with potential interaction between the two features (55). Previous studies have confirmed that the Schwann cell lineage of exogenous nerve fibers has the potential for neurogenesis, while ENCCs cannot be isolated from and cultured ex vivo in the colon wall of patients with total colonic and total intestinal type HSCR due to the absence of exogenous nerve fibers (56). Neurogenesis of Schwann precursors also occurs in the submucosal region in the absence of vagal ENCCs (57). Studies have shown that there is indeed a typical dendritic network composed of neurons and glia in the colon submucosa of HSCR patients, and the co-expression of Tuj1 and HNK1 supports its neural crest origin (58). This finding suggests that exogenous glia derived from the neural crest participate in the construction of the submucosal neural network. Moreover, emerging evidence suggests that enteric glia possess neurogenic potential and exhibit bidirectional interactions with enteric niches (59–61). In addition, Schwann cells have an unstable differentiation state, and under the action of certain exogenous factors, such as nuclear transfer, cell fusion, epigenetic modification, and ectopic gene expression, Schwann cells can dedifferentiate and restore some characteristics of ENCCs (62). Schwann precursors also share many characteristics of their parental ENCCs. Some researchers even refer to Schwann precursors as camouflaged ENCCs (63). In HSCR mice models, neurogenesis originating from Schwann precursors is concentrated in the transition zone and can occur independently of the RET gene (64). Intestinal mucosal biopsy specimens of HSCR patients can be cultured into characteristic neurosphere-like structures, with positive expression of P75 and Nestin, which are “stemness” and can self-proliferate and differentiate into various subtypes of neurons (65). Therefore, full mobilization of exogenous hypertrophic nerve fibers located in the transition zone and Schwann precursors in the submucosal region may be the basis of postnatal neurogenesis and has important reference value. Despite the recent discovery of limited neurogenesis of exogenous nerve fibers and submucosal Schwann precursors, this can only partially restore intestinal function (66, 67). Exploring the potential regulatory mechanisms of dedifferentiation, transdifferentiation, and other exogenous nerve fibers and submucosal Schwann precursors may further stimulate/promote sufficient neurogenesis and provide a solid foundation for the clinical transformation of cell therapy for HSCR.

The narrative of “Rags To Riches” emphasizes the potential of the aberrant enteric niches for transformative discoveries and the importance of perseverance in HSCR research. In the context of HSCR, this metaphor highlights the potential of the aberrant enteric niches for breakthroughs in understanding enteric niches and developing novel therapeutic strategies for HSCR treatment. By embracing the spirit of “Rags To Riches,” researchers can overcome challenges and make significant contributions to the field of HSCR.

## Conclusion

To date, the enteric niches required for embryonic ENS development have not been fully elucidated, and the postnatal microenvironment of ENS maturation is even more complex, which undoubtedly further increases the difficulty of understanding enteric niches. The interaction and underlying mechanisms between ENCCs and their enteric niches remain elusive. Future studies should first “decipher” key changes that lead to abnormalities in enteric niches, “lock” key pathways and potential intervention targets that may lead to abnormal interactions between ENCCs and their niches, and then “correct” key changes to regulate intervention targets, to optimize and construct suitable enteric niches, to regulate the interactions between ENCCs and their niches, and to promote the transplantation efficiency of ENCCs. we underscore the challenges in HSCR treatment highlight the critical role of enteric niches, which encompass not only genomic factors but also microbiota, fibroblasts, extracellular matrix, immune interactions, and muscle cells. While deciphering mutations remains important, a holistic approach is essential to address niche heterogeneity and improve cell transplantation outcomes. Future research must integrate these multifaceted components to develop transformative therapies that restore functional enteric nervous systems.
